# Microarray analysis of formalin-fixed, paraffin-embedded follicular thyroid carcinoma samples from patients who developed postoperative distant metastasis

**DOI:** 10.1186/s13104-020-05080-8

**Published:** 2020-05-13

**Authors:** Tomoo Jikuzono, Tomoko Ishikawa, Mitsuyoshi Hirokawa, Iwao Sugitani, Osamu Ishibashi

**Affiliations:** 1 Laboratory of Biological Macromolecules, Department of Applied Life Sciences, Graduate School of Life & Environmental Sciences, Osaka Prefecture University, 1‑1 Gakuen‑cho, Sakai, 599‑8531 Japan; 2 Department of Endocrine Surgery, Kanaji Thyroid Hospital, 1‑5‑6 Nakazato, Kita‑ku, Tokyo, 114‑0015 Japan; 3 Department of Endocrine Surgery, Nippon Medical School, 1‑1‑5 Sendagi, Bunkyo‑ku, Tokyo, 113‑8602 Japan; 4 Institute for Human Life Innovation, Ochanomizu University, 2‑1‑1 Otsuka, Bunkyo‑ku, Tokyo, 112‑8610 Japan; 5 Department of Diagnostic Pathology and Cytology, Kuma Hospital, 8‑2‑35 Shimoyamate‑dori, Chuo‑ku, Kobe, 650‑0011 Japan

**Keywords:** Minimally invasive follicular thyroid carcinoma, Encapsulated angioinvasive follicular thyroid carcinoma, Microarray analysis, Formalin-fixed and paraffin-embedded samples

## Abstract

**Objective:**

Although follicular thyroid carcinoma (FTC) generally has a good prognosis, it occasionally metastasises, leading to poor prognosis. Unfortunately, minimally invasive FTC (mi-FTC) and encapsulated angioinvasive FTC (ea-FTC) cannot be distinguished cytopathologically from thyroid follicular adenoma (FTA), a benign tumour with a good prognosis. Therefore, a molecular diagnosis to distinguish mi- or ea-FTC from FTA is needed for clinical treatment. Several transcriptomics/proteomics studies have searched for FTC biomarkers. However, the results of these studies were not consistent, which could be partly explained by inaccurate diagnosis of the specimens analysed.

**Data description:**

We conducted a microarray-based genome-wide transcriptome analysis using formalin-fixed paraffin-embedded mi- or ea-FTC specimens from patients who developed distant metastasis up to 10 years postoperatively, which ensured the accuracy of diagnosis.

## Objective

Follicular thyroid carcinoma (FTC) and papillary thyroid carcinoma are major histological types of thyroid carcinoma. Until recently, FTC was subdivided into minimally invasive FTC (mi-FTC) and widely invasive FTC, based on the World Health Organization (WHO) classification (3^rd^ edition). The recent 4th edition [1] added a new FTC subtype, designated encapsulated angioinvasive FTC (ea-FTC), which can be differentiated from mi-FTC by its limited vascular invasion. Both FTCs have good long-term outcomes; however, these FTCs occasionally metastasise to lung and bone, and exhibit a poor prognosis [2]. It is difficult to distinguish metastatic and non-metastatic mi/ea-FTCs pathologically [3, 4]. Thus, prognostic biomarkers for prediction of the risk of metastasis for patients diagnosed with mi- or ea-FTC would help to determine the postoperative treatment of these FTCs.

Post-genome studies have revealed that numerous non-coding RNAs (ncRNAs) are transcribed from the human genome [5]. Interestingly, aberrant expression patterns of some ncRNAs are associated with cancer [6, 7]; this suggests that ncRNAs are promising diagnostic biomarkers for cancer. While previous studies have focused on the expression of coding RNAs and microRNAs [8–11], there is little information regarding expression of long ncRNAs in FTC. Therefore, this study examined possible RNA biomarkers for the molecular diagnosis of metastatic mi/ea-FTC.

The specimens used in this study were equivalent to ea-FTC or mi-FTC in postoperative pathological examination. To increase the accuracy of analysis, we selected patients with a diagnosis of metastatic mi/ea-FTC that had been established using the following criteria: i) routine thyroglobulin testing and neck ultrasonography for ≥ 10 years, and ii) recognition of distant metastasis after the initial operation. RNA was extracted from formalin-fixed paraffin-embedded (FFPE) specimens of the tumour and adjacent non-tumour tissues from patients with mi/ea-FTC. The RNA samples were then subjected to genome-wide transcriptome analysis, which enabled profiling of transcripts including ncRNAs.

## Data description

Table 1 summarises the data reported herein.

**Table 1:** Overview of data files/data sets

Label	Name of data file/data set	File types (file extension)	Data repository and identifier (DOI or accession number)
Data file 1	GSM3031433_701012-009_Clariom_D_Human_.CEL.gz	CEL file	https://identifiers.org/geo:GSM3031433 [13]
Data file2	GSM3031434_701012-002_Clariom_D_Human_.CEL.gz	CEL file	https://identifiers.org/geo:GSM3031434 [14]
Data file 3	GSM3031435_701012-003_Clariom_D_Human_.CEL.gz	CEL file	https://identifiers.org/geo:GSM3031435 [15]
Data file 4	GSM3031436_701012-004_Clariom_D_Human_.CEL.gz	CEL file	https://identifiers.org/geo:GSM3031436 [16]
Data file 5	GSM3031437_701012-005_Clariom_D_Human_.CEL.gz	CEL file	https://identifiers.org/geo:GSM3031437 [17]
Data file 6	GSM3031438_701012-010_Clariom_D_Human_.CEL.gz	CEL file	https://identifiers.org/geo:GSM3031438 [18]
Data file 7	Clinical features	Spreadsheet (.xlsx)	Mendeley Data (http://dx.doi.org/10.17632/vjr6z8kw77.7) [19]
Data file 8	Size distribution of total RNA	Image (.pdf)	Mendeley Data (http://dx.doi.org/10.17632/3kx9gzj2yt.3) [20]
Data file 9	Size distribution of fragmented cDNAs	Image (.pdf)	Mendeley Data (http://dx.doi.org/10.17632/n5cjz2z26f.3) [21]
Data file 10	Scatterplots_carcinoma vs normal	Image (.pdf)	Mendeley Data (http://dx.doi.org/10.17632/dhv8767r3z.4) [22]
Data file 11	Normalised signal values	Spreadsheet (.xlsx)	Mendeley Data (http://dx.doi.org/10.17632/mwgjj6xszj.5) [23]

In this study, specimens from three patients who underwent surgery at Kuma Hospital (Hyogo, Japan) were selected for analysis: one had diagnostic features of mi-FTC and two had features of ea-FTC. Data file 7 summarises the clinical findings of these patients. The primary surgical specimens were evaluated histopathologically in accordance with WHO criteria. This study was conducted in accordance with the principles of the 1975 Declaration of Helsinki; informed consent was obtained from each patient. Archival mi/ea-FTC FFPE samples were processed into 20-μm sections; regions containing carcinoma or non-carcinoma tissues in each section were isolated separately. Total RNA was extracted from these samples using NucleoSpin^®^ totalRNA FFPE XS (Takara Bio, Kusatsu, Japan). RNA concentrations were measured spectrophotometrically (DS-11 NanoPad; DeNovix, Wilmington, DE, USA). The size distribution of total RNA was analysed using an Agilent 2100 Bioanalyzer (Agilent, Santa Clara, CA, USA) (Data file 8).

An Affymetrix Clariom D Assay (Thermo Fisher Scientific), a next-generation microarray for transcriptome profiling, was used for genome-wide transcriptome analysis to increase the possibility of biomarker discovery. Labelled targets were prepared from RNA samples using the GeneChip^®^ WT Pico Reagent Kit (Thermo Fisher Scientific) with slight modification. Briefly, 50 ng of total RNA from each sample were reverse transcribed and subjected to polymerase chain reaction to synthesise T7 promoter-tagged double stranded cDNA. The cDNA was then subjected to in vitro transcription to synthesise complementary RNA; 20 ng of complementary RNA was reverse-transcribed using random primers for sense strand cDNA synthesis. After removal of template RNA using RNase H, 5.5 or 3.2 μg sense strand cDNA were digested with uracil-DNA glycosidase into 40–70-nt fragments. Successful fragmentation was confirmed using an Agilent 2100 Bioanalyzer (Agilent) (Data file 9). The fragmented cDNA was then biotin-labelled with terminal deoxynucleotidyl transferase and subjected to hybridisation performed in accordance with the GeneChip^®^ WT Pico Reagent Kit manual. The Clariom D array was then processed through the automatic washing step using the GeneChip^®^ Hybridisation, Wash, and Stain Kit (Thermo Fisher Scientific) and Fluidics Station 450 (Thermo Fisher Scientific). Hybridised targets on the array were stained with streptavidin–phycoerythrin provided in the kit and detected using Scanner 3000 7G (Thermo Fisher Scientific).

Raw data (CEL files) were produced for the six samples using Affymetrix GeneChip Command Console Software and processed using Affymetrix Expression Console Software. The CEL files are registered under Gene Expression Omnibus (GEO) accession no. 701012. A detection call algorithm was used to filter and remove missing expression values based on absent/present calls. Using this algorithm, present, marginal, or absent calls were obtained for each probe set in each array. A scaling factor was applied to the normalised data from the CEL files to bring the average intensity for all probes on the array to 500, generating CHP files using Microarray Suite 5 software. To compare gene expression, data assigned to absent calls were omitted.

Data file 10 shows scatterplots of the correlation of signal values between carcinoma and non-carcinoma samples. Normalised signal values for individual genes are listed in Data file 11.

## Limitations

Herein, we provide information regarding a mi/ea-FTC microarray dataset, which will help to identify prognostic biomarkers for predicting high risk of metastasis among patients diagnosed with mi/ea-FTC, following the initial operation. Furthermore, such biomarkers may be used to diagnose FTC in thyroid specimens obtained by fine-needle aspiration biopsy for liquid-based cytology [9]. However, we used total RNA isolated from FFPE samples, which is known to be highly degraded [12]. Although the Clariom D Assay was designed for application to analyses using such low-quality RNA samples, the data should be verified carefully. Transcriptome analysis of FTA samples should be performed in a similar manner for comparison.

## Data Availability

The raw microarray data described in this article can be freely and openly accessed on the Gene Expression Omnibus (GEO) database under the accession number GSE111455 [13–18]. The image and table data described in this article are available from Mendeley Data [19–23]. Please see Table 1 for details and links to the data.

## Abbreviations

ea-FTC: Encapsulated angioinvasive follicular thyroid carcinoma
FFPE: Formalin-fixed and paraffin-embedded
FTA: Thyroid follicular adenoma
FTC: Follicular thyroid carcinoma
GEO: Gene expression omnibus
mi-FTC: Minimally invasive follicular thyroid carcinoma
WHO: The World Health Organization
